# Identifying metabolic features of colorectal cancer liability using Mendelian randomization

**DOI:** 10.1101/2023.03.10.23287084

**Published:** 2023-03-10

**Authors:** Caroline J. Bull, Emma Hazelwood, Joshua A. Bell, Vanessa Y. Tan, Andrei-Emil Constantinescu, Maria Carolina Borges, Danny N. Legge, Kimberly Burrows, Jeroen R. Huyghe, Hermann Brenner, Sergi Castellví-Bel, Andrew T Chan, Sun-Seog Kweon, Loic Le Marchand, Li Li, Iona Cheng, Rish K. Pai, Jane C. Figueiredo, Neil Murphy, Marc J. Gunter, Nicholas J. Timpson, Emma E. Vincent

**Affiliations:** 1 MRC Integrative Epidemiology Unit at the University of Bristol, Bristol, UK; 2 Population Health Sciences, Bristol Medical School, University of Bristol, Bristol, UK; 3 Translational Health Sciences, Bristol Medical School, University of Bristol, UK; 4 Public Health Sciences Division, Fred Hutchinson Cancer Center, Seattle, Washington, USA; 5 Division of Clinical Epidemiology and Aging Research, German Cancer Research Center (DKFZ), Heidelberg, Germany; 6 Division of Preventive Oncology, German Cancer Research Center (DKFZ) and National Center for Tumor Diseases (NCT), Heidelberg, Germany; 7 German Cancer Consortium (DKTK), German Cancer Research Center (DKFZ), Heidelberg, Germany; 8 Gastroenterology Department, Hospital Clínic, Institut d’Investigacions Biomèdiques August Pi i Sunyer (IDIBAPS), Centro de Investigación Biomédica en Red de Enfermedades Hepáticas y Digestivas (CIBEREHD), University of Barcelona, Barcelona, Spain; 9 Division of Gastroenterology, Massachusetts General Hospital and Harvard Medical School, Boston, Massachusetts, USA; 10 Channing Division of Network Medicine, Brigham and Women’s Hospital and Harvard Medical School, Boston, Massachusetts, USA; 11 Clinical and Translational Epidemiology Unit, Massachusetts General Hospital and Harvard Medical School, Boston, Massachusetts, USA; 12 Broad Institute of Harvard and MIT, Cambridge, Massachusetts, USA; 13 Department of Epidemiology, Harvard T.H. Chan School of Public Health, Harvard University, Boston, Massachusetts, USA; 14 Department of Immunology and Infectious Diseases, Harvard T.H. Chan School of Public Health, Harvard University, Boston, Massachusetts, USA; 15 Department of Preventive Medicine, Chonnam National University Medical School, Gwangju, Korea; 16 Jeonnam Regional Cancer Center, Chonnam National University Hwasun Hospital, Hwasun, Korea; 17 University of Hawaii Cancer Center, Honolulu, Hawaii, USA; 18 Department of Family Medicine, University of Virginia, Charlottesville, Virginia, USA; 19 Department of Epidemiology and Biostatistics, University of California, San Francisco, San Francisco, California, USA; 20 University of California, San Francisco Helen Diller Family Comprehensive Cancer Center, San Francisco, San Francisco, California, USA; 21 Department of Pathology and Laboratory Medicine, Mayo Clinic, Arizona, Scottsdale, Arizona, USA; 22 Department of Medicine, Samuel Oschin Comprehensive Cancer Institute, Cedars-Sinai Medical Center, Los Angeles, California, USA; 23 Department of Epidemiology and Biostatistics, School of Public Health, Imperial College London, United Kingdom; 24 Nutrition and Metabolism Branch, International Agency for Research on Cancer, Lyon, France

**Keywords:** Colorectal cancer, Metabolism, Genetics, NMR, Epidemiology, Mendelian randomization, ALSPAC, UK Biobank, GECCO, CCTS, CCFR

## Abstract

Recognizing the early signs of cancer risk is vital for informing prevention, early detection, and survival. To investigate whether changes in circulating metabolites characterise the early stages of colorectal cancer (CRC) development, we examined associations between a genetic risk score (GRS) associated with CRC liability (72 single nucleotide polymorphisms) and 231 circulating metabolites measured by nuclear magnetic resonance spectroscopy in the Avon Longitudinal Study of Parents and Children (N=6,221). Linear regression models were applied to examine associations between genetic liability to colorectal cancer and circulating metabolites measured in the same individuals at age 8, 16, 18 and 25 years. The GRS for CRC was associated with up to 28% of the circulating metabolites at FDR-P<0.05 across all time points, particularly with higher fatty acids and very-low- and low-density lipoprotein subclass lipids. Two-sample reverse Mendelian randomization (MR) analyses investigating CRC liability (52,775 cases, 45,940 controls) and metabolites measured in a random subset of UK Biobank participants (N=118,466, median age 58y) revealed broadly consistent effect estimates with the GRS analysis. In conventional (forward) MR analyses, genetically predicted polyunsaturated fatty acid concentrations were most strongly associated with higher CRC risk. These analyses suggest that higher genetic liability to CRC can cause early alterations in systemic metabolism, and suggest that fatty acids may play an important role in CRC development.

## Background

Colorectal cancer (CRC) is the third most frequently diagnosed cancer worldwide and the fourth most common cause of death from cancer.^1,2^ There is a genetic component to risk of the disease, which is thought to explain up to 35% of variability in CRC risk.^3–5^ In addition, modifiable lifestyle factors, including obesity, consumption of processed meat, and alcohol are thought to increase CRC risk.^2,6–9^ However, the underlying biological pathways remain unclear, which limits targeted prevention strategies. Whilst CRC has higher mortality rates when diagnosed at later stages, early-stage CRC or precancerous lesions are largely treatable, meaning colorectal cancer screening programmes have the potential to be highly effective.^10,11^ Due to the lack of known predictive biomarkers for CRC, wide-scale screening (if implemented at all) is expensive and often targeted crudely by age range. Identifying biomarkers predictive of CRC, or with causal roles in disease development, is therefore vital.

One potential source of biomarkers for CRC risk is the circulating metabolome, which offers a dynamic insight into cellular processes and disease states. It is increasingly clear from mechanistic studies that both systemic and intracellular tumour metabolism play an important role in CRC development and progression.^12,13^ Interestingly, several major risk factors for CRC are known to have profound effects on metabolism.^14^ For instance, obesity has been shown via conventional observational and Mendelian randomization (MR) analyses to strongly alter circulating metabolite levels.^9,15–17^ This suggests that the circulating metabolome may play a mediating role in the relationship between at least some common risk factors, such as obesity, and CRC – or at least might be a useful biomarker for disease or intermediates thereof. Investigating the relationship between CRC and circulating metabolites may therefore provide powerful insights into the causal pathways underlying disease risk, or alternatively may be valuable in prediction and early diagnosis.

MR is a genetic epidemiological approach used to evaluate causal relationships between traits.^18,19^ This method uses genetic variation as a proxy measure for traits in an instrumental variable framework to assess the causal relevance of the traits in disease development. As germline genetic variants are theoretically randomised between generations and fixed at conception, this approach should be less prone to bias and confounding than conventional analyses undertaken in an observational context. Conventionally, MR is used to investigate the effect of an exposure on a disease outcome. In reverse MR, genetic instruments proxy the association between liability to a disease and other traits.^20^ This approach can identify biomarkers which cause the disease, are predictive for the disease, or have diagnostic potential.^20^ Given the suspected importance of the circulating metabolome in CRC development, employing both reverse MR and conventional forward MR for metabolites in the same study may be an efficient approach for revealing causal and predictive biomarkers for CRC. Although previous observational studies have investigated associations between the circulating metabolome and CRC risk, these studies may have been influenced by confounding bias which should be less relevant to MR analyses.^21–30^ Additionally, these studies focussed on adults, who commonly take medications which may confound metabolite associations, further complicating interpretations.

Here, we applied a reverse MR framework to identify circulating metabolites which are associated with CRC liability across different stages of the early life course (spanning childhood to young adulthood, when use of medications and CRC are both rare) using data from a birth cohort study. We then attempted to replicate these results using reverse two-sample MR in an independent cohort of middle-aged adults (UK Biobank). We then performed conventional ‘forward’ MR of metabolites onto CRC risk using large-scale cancer consortia data to identify metabolites which may have a causal role in CRC development.

## Methods

### Study populations

This study uses data from 2 cohort studies: the Avon Longitudinal Study of Parents and Children (ALSPAC) offspring (generation 1) cohort (individual-level data) and the UK Biobank cohort (summary-level data); plus summary-level data from a genome-wide association study (GWAS) meta-analysis of CRC comprising the Genetics and Epidemiology of Colorectal Cancer Consortium (GECCO), Colorectal Transdisciplinary Study (CORECT), and Colon Cancer Family Registry (CCFR).

ALSPAC is a population-based birth cohort study in which 14,541 pregnant women with an expected delivery date between 1 April 1991 and 31 December 1992 were recruited from the former Avon County of southwest England.^31^ Since then, 13,988 offspring alive at one year have been followed repeatedly with questionnaire- and clinic-based assessments.^32,33^ Study data were collected and managed using REDCap electronic data capture tools hosted at the University of Bristol.^34^ REDCap (Research Electronic Data Capture) is a secure, web-based software platform designed to support data capture for research studies. Offspring genotype was assessed using the Illumina HumanHap550 quad chip platform. Quality control measures included exclusion of participants with sex mismatch, minimal or excessive heterozygosity, disproportionately missing data, insufficient sample replication, cryptic relatedness, and non-European ancestry. Imputation was performed using the Haplotype Reference Consortium (HRC) panel. Offspring were considered for the current analyses if they had no older siblings in ALSPAC (203 excluded) and were of white ethnicity (based on reports by parents, 604 excluded) to reduce the potential for confounding by genotype. Written informed consent was provided and ethical approval was obtained from the ALSPAC Law and Ethics Committee and the local research ethics committee. Consent for biological samples has been collected in accordance with the Human Tissue Act (2004). Informed consent for the use of data collected via questionnaires and clinics was obtained from participants following the recommendations of the ALSPAC Ethics and Law Committee at the time. The study website contains details of all available data through a fully searchable data dictionary and variable search tool (http://www.bristol.ac.uk/alspac/researchers/our-data/).

UK Biobank is a population-based cohort study based in 22 centres across the UK.^35^ The cohort is made up of around 500,000 adults aged 40–80 years old, who were enrolled between 2006 and 2010. Genotyping data is available for 488,377 participants.^36^ Participants were genotyped using one of two arrays – either the Applied Biosystems UK BiLEVE Axiom Array by Affymetrix (now part of Thermo Fisher Scientific), or the closely related Applied Biosystems UK Biobank Axiom Array. Approaches based on Principal Component Analysis (PCA) were used to account for population structure. Individuals were excluded: if reported sex differed from inferred sex based on genotyping data; if they had sex chromosome karyotypes which were not XX or XY; if they were outliers in terms of heterozygosity and missing rates; or if they had high relatedness to another participant. Multiallelic SNPs or those with a minor allele frequency of below 1% were removed. Imputation was performed using the UK10K haplotype and HRC reference panels.

The GWAS meta-analysis for CRC included up to 52,775 cases and 45,940 controls.^37,38^ This sample excluded cases and controls from UK Biobank to avoid potential bias due to sample overlap which may be problematic in MR analyses.^39^ Cases were diagnosed by a physician and recorded overall and by site (colon, 28,736 cases; proximal colon, 14,416 cases; distal colon, 12,879 cases; and rectal, 14,150 cases). Colon cancer included proximal colon (any primary tumour arising in the cecum, ascending colon, hepatic flexure, or transverse colon), distal colon (any primary tumour arising in the pleenic flexure, descending colon or sigmoid colon), and colon cases with unspecified site. Rectal cancer included any primary tumour arising in the rectum or rectosigmoid junction.^37^ Approximately 92% of participants in the overall CRC GWAS were white-European (~8% were East Asian). All participants included in site-specific CRC analyses were of European ancestry. Imputation was performed using the Michigan imputation server and HRC r1.0 reference panel. Regression models were further adjusted for age, sex, genotyping platform, and genomic principal components as described previously.^37^ Ethics were approved by respective institutional review boards.

### Assessment of CRC genetic liability

Genetic liability to CRC was based on single nucleotide polymorphisms (SNPs) associated with CRC case status at genome-wide significance (P<5×10^−8^). 108 independent SNPs reported by two major GWAS meta-analyses were eligible for inclusion in a CRC genetic risk score (GRS).^37,40^ The set of SNPs was filtered, excluding 36 SNPs that were in linkage disequilibrium based on R^2^>0.001 using the TwoSampleMR package (SNPs with the lowest P-values were retained).^41^ This left 72 SNPs independently associated with CRC (Supplementary Table (ST) 1), 65 of which were available in imputed ALSPAC genotype data post quality control. As GWAS of site-specific CRC have identified marked heterogeneity,^42^ GRS describing site-specific CRCs were constructed for sensitivity analyses using the same process outlined above. The GRS for colon cancer, rectal cancer, proximal colon cancer and distal colon cancer were comprised of 38, 25, 20 and 24 variants, respectively (ST1). For overall CRC and site-specific CRC analyses, sensitivity analyses excluding any SNPs in the FADS cluster (i.e. within the gene regions of *FADS1*, *FADS2*, or *FADS3*) (ST1) were performed given a likely role for these SNPs in influencing circulating metabolite levels directly, in particular via lipid metabolism (i.e., not primarily due to CRC).^43–49^

### Assessment of circulating metabolites

Circulating metabolite measures were drawn from ALSPAC and UK Biobank using the same targeted metabolomics platform. In ALSPAC, participants provided non-fasting blood samples during a clinic visit while aged approximately 8y, and fasting blood samples from clinic visits while aged approximately 16y, 18y, and 25y. Proton nuclear magnetic resonance (^1^H-NMR) spectroscopy was performed on Ethylenediaminetetraacetic acid (EDTA) plasma (stored at or below −70 degrees Celsius pre-processing) to quantify a maximum of 231 metabolites.^50^ Quantified metabolites included the cholesterol and triglyceride content of lipoprotein particles; the concentrations and diameter/size of these particles; apolipoprotein B and apolipoprotein A-1 concentrations; as well as fatty acids and their ratios to total fatty acid concentration, branched chain and aromatic amino acids, glucose and pre-glycaemic factors including lactate and citrate, fluid balance factors including albumin and creatinine, and the inflammatory marker glycoprotein acetyls (GlycA). In UK Biobank, EDTA plasma samples from 117,121 participants, a random subset of the original ~500,000 who provided samples at assessment centres between 2006 and 2013, were analysed between 2019 and 2020 for levels of 249 metabolic traits (168 concentrations plus 81 ratios) using the same high-throughput ^1^H-NMR platform. Data pre-processing and QC steps are described previously.^50–52^ To allow comparability between MR and GRS estimates all metabolite measures were standardised and normalised using rank-based inverse normal transformation. For descriptive purposes in ALSPAC, body mass index (BMI) was calculated at each time point as weight (kg) divided by squared height (m^2^) based on clinic measures of weight to the nearest 0.1 kg using a Tanita scale and height measured in light clothing without shoes to the nearest 0.1 cm using a Harpenden stadiometer.

CRC liability variants were combined into a GRS using PLINK 1.9, specifying the effect (risk raising) allele and coefficient (logOR) with estimates from the CRC GWAS used as external weights.^37,40^ GRSs were calculated as the number of effect alleles (or dosages if imputed) at each SNP (0, 1, or 2) multiplied by its weighting, summing these, and dividing by the total number of SNPs used. Z-scores of GRS variables were calculated to standardize scoring.

### Statistical approach

**Figure 1:**
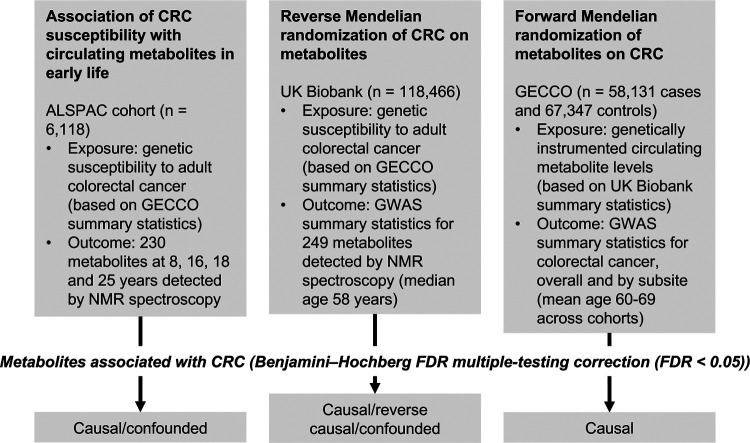
Study design. First, linear regression models were used to examine the relationship between genetic susceptibility to adult colorectal cancer and circulating metabolites measured in ALSPAC participants at age 8, 16, 18 and 25 years. Next, we performed a reverse Mendelian randomization analysis to identify metabolites influenced by CRC susceptibility in an independent population of adults. Finally, we performed a conventional (forward) Mendelian randomization analysis of circulating metabolites on CRC to identify metabolites causally associated with CRC risk. Consistent evidence across all three methodological approaches was interpreted to indicate a causal role for a given metabolite in CRC etiology.

An overview of the study design is presented in Figure 1. To estimate the effect of increased genetic liability to CRC on circulating metabolites we conducted a GRS analysis in ALSPAC and reverse two-sample MR analyses in UK Biobank. Estimates were interpreted within a ‘reverse MR’ framework,^53^ wherein results are taken to reflect ‘metabolic features’ of CRC liability which could capture causal or predictive metabolite-disease associations. To clarify the direction of metabolite-CRC associations, we additionally performed conventional ‘forward’ two-sample MR analyses to estimate the effect of circulating metabolites on CRC risk using large-scale GWAS data on metabolites and CRC.

#### 1. Associations of CRC liability with circulating metabolites in early life

Separate linear regression models with robust standard errors were used to estimate coefficients and 95% confidence intervals for associations of GRSs with each metabolite as a dependent variable measured on the same individuals at age 8y, 16y, 18y, and 25y, adjusted for sex and age at the time of metabolite assessment. To aid interpretations, estimates were multiplied by 0.693 (log_e_2) to reflect SD-unit differences in metabolites per doubling of genetic liability to CRC.^54^ The Benjamini-Hochberg method was used to adjust P-values for multiple testing and an adjusted P-value of <0.05 was used as a heuristic for evidence for association given current sample sizes.^55^

#### 2. Reverse MR of the effects of CRC liability on circulating metabolites in middle adulthood

“Reverse” MR analyses^53^ were conducted using UK Biobank for outcome datasets in two sample MR to examine the effect of CRC liability on circulating metabolites. SNP-outcome (metabolite) estimates were obtained from a GWAS of metabolites in UK Biobank.^56,57^ Prior to GWAS, all metabolite measures were standardised and normalised using rank-based inverse normal transformation. Genetic association data for metabolites were retrieved using the MRC IEU UK Biobank GWAS pipeline.^58^ Full summary statistics are available via the IEU Open GWAS project.^53,59^ Up to 3 statistical methods were used to generate reverse MR estimates of the effect of CRC liability on circulating metabolites using the TwoSampleMR package^60^: random-effects inverse variance weighted (IVW), weighted-median, and weighted-mode, which each make differing assumptions about directional pleiotropy and SNP heterogeneity.^61–63^ As above, estimates were multiplied by 0.693 (log_e_2) to reflect SD-unit differences in metabolites per doubling of genetic liability to CRC.^64^

#### 3. Forward MR of the effects of metabolites on CRC

Forward MR analyses were conducted using summary statistics from UK Biobank for the same NMR-measured metabolites (SNP-exposure) and from GECCO/CORECT/CCFR as outlined above (SNP-outcome). We identified SNPs that were independently associated (R^2^<0.001 and P<5×10^−8^) with metabolites from a GWAS of 249 metabolites in UK Biobank described above. As before, we used up to 3 statistical methods to generate MR estimates of the effect of circulating metabolites on CRC risk (overall and site-specific): random-effects IVW, weighted-median, and weighted-mode. The Benjamini-Hochberg method was used to adjust P-values for multiple testing and an adjusted P-value of <0.05 was used as a heuristic for nominal evidence for a causal effect.^55^ MR outputs are beta coefficients representing the logOR for CRC per SD higher metabolite, exponentiated to reflect the OR for CRC per SD metabolite.

MR analyses were performed in R version 4.0.3.^65^ and GRS analyses in Stata 16.1 (StataCorp, College Station, Texas, USA). The ggforestplot R package was used to generate results visualisations.^66^

## Results

### Associations of CRC liability with circulating metabolites in early life

At the time the ALSPAC blood samples were taken, the mean age of participants was 7.5y (N=4,767), 15.5y (N=2,930), 17.8y (N=2,613), and 24.5y (N=2,559) for the childhood, early adolescence, late adolescence and young adulthood time points respectively. The proportion of participants which were male were 50.5%, 47.4%, 44.5%, and 39.1% and mean BMI was 16.2, 21.4, 22.7, and 24.8 kg/m^2^ for each time point respectively. The socio-demographic profile of ALSPAC offspring participants has been reported previously.^67^ Mean and standard deviation (SD) values for metabolites on each measurement occasion in ALSPAC are shown in ST2.

**Figure 2:**
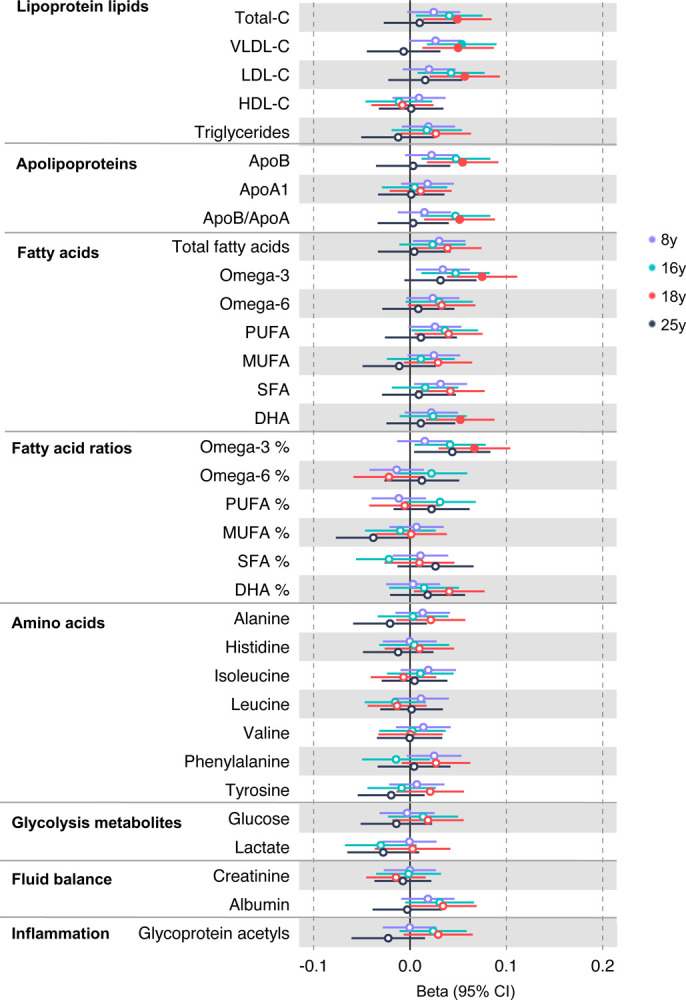
Associations of genetic liability to adult colorectal cancer (based on a 72 SNP genetic risk score) with clinically validated metabolic traits at different early life stages among ALSPAC offspring (age 8y, 16y, 18y, and 25y). Estimates shown are beta coefficients representing the SD difference in metabolic trait per doubling of genetic liability to colorectal cancer (purple, 8y; turquoise, 16y; red, 18y; black, 25y). Filled point estimates are those that pass a Benjamini–Hochberg FDR multiple-testing correction (FDR < 0.05).

In the GRS analysis, there was no strong evidence of association of CRC liability with metabolites at age 8y (ST3). At age 16y, there was evidence for association with several lipid traits including higher cholesteryl esters to total lipids ratio in large low-density lipoprotein (LDL) (SD change per SD higher CRC liability = 0.060, 95% CI = 0.024 to 0.096) and higher cholesterol in very small very low-density lipoprotein (VLDL) (SD change per SD higher liability = 0.06, 95% CI = 0.03 to 0.10). There was strong evidence for association with several traits at age 18y including higher non-high-density lipoprotein (non-HDL) lipids, e.g., a 1 SD higher CRC liability was associated with higher levels of total cholesterol (SD change = 0.05 95% CI = 0.01 to 0.09), VLDL-cholesterol (SD change = 0.05, 95% CI = 0.01 to 0.09), LDL-cholesterol (SD change = 0.06, 95% CI = 0.02 to 0.09)), apolipoproteins (apolipoprotein B (SD change = 0.06, 95% CI = 0.02 to 0.09)), and fatty acids (omega-3 (SD change = 0.08, 95% CI = 0.04 to 0.11), docosahexaenoic acid (DHA) (SD change = 0.05, 95% CI = 0.02 to 0.09)) (ST3). Figure 2 shows results for all clinically validated metabolites. At age 25y, there was no strong evidence of association of CRC liability with metabolites. In anatomical site-specific analyses, there was strong evidence for association of liability to colon cancer with omega-3 (SD change = 0.07, 95% CI = 0.03 to 0.11) and DHA (SD change = 0.07, 95% CI = 0.03 to 0.10) at age 18y. There was little evidence for any associations at any other CRC site or age (ST3, Supplementary Figure (SF)1–4). When SNPs in the FADS cluster gene regions were excluded due to possible horizontal pleiotropy given the role of FADS in lipid metabolism, there was a reduction in strength of evidence for an association of liability to CRC with any metabolite measured, although estimates were in a largely consistent direction with the prior analysis (ST4, SF5–6).

### Reverse MR of the effects of CRC liability on circulating metabolites in middle adulthood

**Figure 3:**
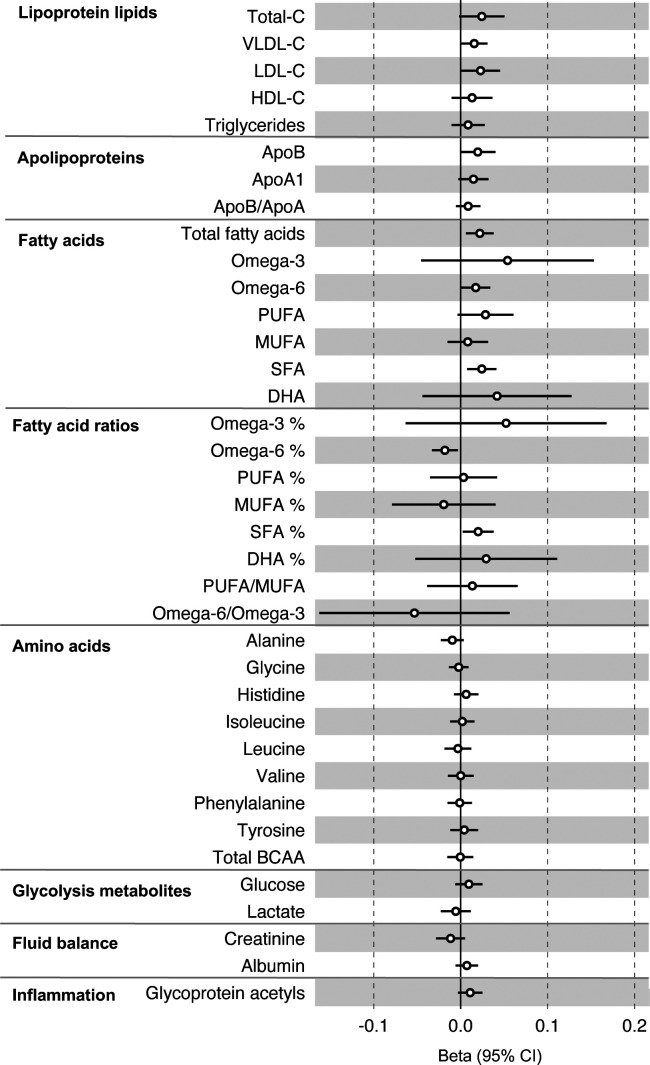
Associations of genetic liability to colorectal cancer with clinically validated metabolic traits in an independent sample of adults based on reverse two sample Mendelian randomization analyses. Estimates shown are beta coefficients representing the SD-unit difference in metabolic trait per doubling of liability to colorectal cancer. Filled point estimates are those that pass a Benjamini–Hochberg FDR multiple-testing correction (FDR < 0.05).

All instrument sets from the reverse MR analysis had an F-statistic greater than 10 (minimum F-statistic = 36, median = 40), suggesting our analyses did not suffer from weak instrument bias (ST5). There was little evidence of an association of CRC liability (overall or by anatomical site) on any of the circulating metabolites investigated, including when the SNP in the *FADS* gene region was excluded, based on our pre-determined cut-off of FDR-P < 0.05; however, the direction of effect estimates was largely consistent with those seen in ALSPAC GRS analyses, with higher CRC liability weakly associated with higher non-HDLs, lipoproteins and fatty acid levels (SF7–9, ST6–7). Figure 3 shows the results for clinically validated metabolites. In subsite stratified analyses, there was strong evidence for a causal effect of genetic liability to proximal colon cancer on several traits, including total fatty acids (SD change per doubling of liability = 0.02, 95% CI = 0.01 to 0.04) and omega-6 fatty acids (SD change per doubling of liability = 0.03, 95% CI = 0.01 to 0.05).

### Forward MR for the effects of metabolites on CRC risk

**Figure 4:**
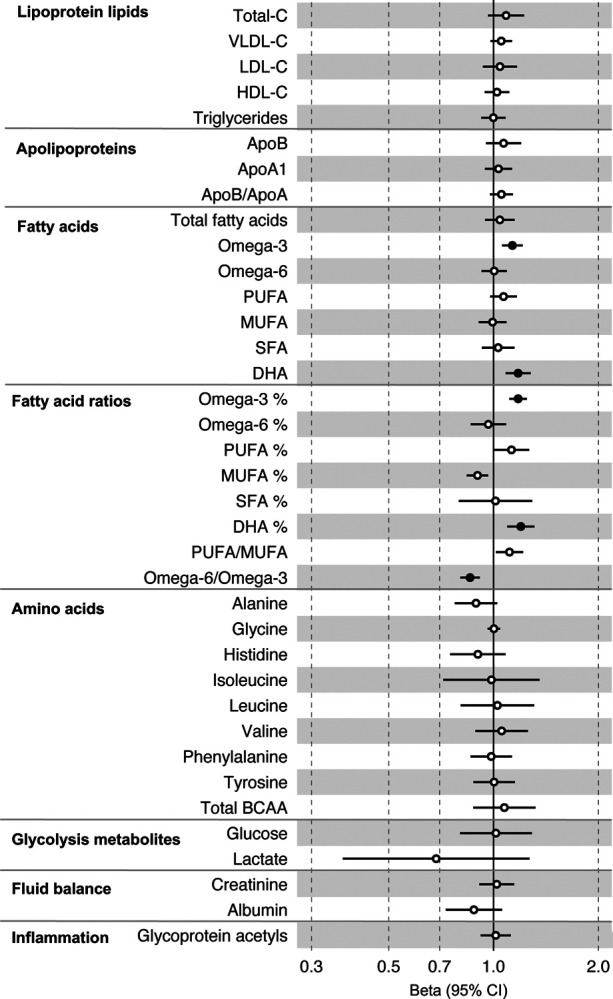
Associations of clinically validated metabolites with colorectal cancer based on conventional (forward) two sample Mendelian randomization analyses. Estimates shown are beta coefficients representing the logOR for colorectal cancer per SD metabolite. Filled point estimates are those that pass a Benjamini–Hochberg FDR multiple-testing correction (FDR < 0.05).

All instrument sets from the forward MR analysis had an F-statistic greater than 10 (minimum F-statistic = 54, median = 141), suggesting that our analyses were unlikely to suffer from weak instrument bias (ST8–9). There was strong evidence for an effect of several fatty acid traits on overall CRC risk, including of omega-3 fatty acids (CRC OR = 1.13, 95% CI = 1.06 to 1.21), DHA (OR CRC = 1.76, 95% CI = 1.08 to 1.28), ratio of omega-3 fatty acids to total fatty acids (OR CRC = 1.18, 95% CI = 1.11 to 1.25), ratio of DHA to total fatty acids (CRC OR = 1.20, 95% CI = 1.10 to 1.31), and ratio of omega-6 fatty acids to omega-3 fatty acids (CRC OR = 0.86, 95% CI = 0.80 to 9.13) (ST10, Figure 4). These estimates were overlapping with variable precision in MR sensitivity models. When SNPs in the *FADS* gene region were excluded, there was little evidence for a causal effect of any metabolite investigated on CRC risk based on the predetermined FDR-P cut of off < 0.05, although the directions of effect estimates were consistent with previous analyses (ST11, SF11–12).

In anatomical subtype stratified analyses evidence was strongest for an effect of fatty acid traits on higher CRC risk, and this appeared specific to the distal colon, e.g., omega-3 (distal CRC OR = 1.20, 95% CI = 1.09 to 1.32), and ratio of DHA to total fatty acids (distal colon OR = 1.29, 95% CI = 1.16 to 1.43). There was also evidence of a negative effect of ratio of omega-6 to omega-3 fatty acids (distal CRC OR = 0.80, 95% CI = 0.74 to 0.88) and a positive effect of ratio of omega-3 fatty acids to total fatty acids (distal CRC = 1.24, 95% CI = 1.15 to 1.35; seen also for proximal CRC OR = 1.15, 95% CI = 1.07 to 1.23) (ST10, SF10). These estimates were also directionally consistent in MR sensitivity models.

## Discussion

Here, we used a reverse MR framework to identify circulating metabolites which are associated with genetic CRC liability across different stages of the early life course and attempted to replicate results in an independent cohort of middle-aged adults. We then performed forward MR to characterise the causal direction of the relationship between metabolites and CRC. Our GRS analysis provided evidence for an association of genetic liability to CRC with higher circulating levels of lipoprotein lipids (including total cholesterol, VLDL-cholesterol, and LDL-cholesterol), apolipoproteins (including apolipoprotein B), and fatty acids (including omega-3 and DHA) in young adults. These results were largely consistent in direction (though smaller in magnitude and weaker in strength of evidence) in a two-sample MR analysis in an independent cohort of middle-aged adults. Results were attenuated, but consistent in direction, when potentially pleiotropic SNPs in the *FADS* gene regions were excluded. However, it should be noted that use of a narrow window for exclusion based on being within one of the three *FADS* genes may mean that some pleiotropic SNPs remain. Our subsequent forward MR analysis highlighted polyunsaturated fatty acids as potentially having a causal role in the development of CRC.

Our analyses highlight a potentially important role of polyunsaturated fatty acids in colorectal cancer liability. However, these analyses may be biased by substantial genetic pleiotropy among fatty acid traits. SNPs which are associated with levels of one fatty acid are generally associated with levels of many more fatty acid (and non-fatty acid) traits.^68,69^ For instance, genetic instruments within the FADS cluster of genes will likely affect both omega-3 and omega-6 fatty acids, given *FADS1* and *FADS2* encode enzymes which catalyse the conversion of both from shorter chain into longer chain fatty acids.^69^ In addition, the NMR metabolomics platform utilised in the analyses outlined here has limited coverage of fatty acids, meaning many putative causal metabolites for CRC, for example arachidonic acid, could not be investigated. Therefore, although our results indicate that polyunsaturated fatty acids may be important in colorectal cancer risk, given the pleiotropic nature of the fatty acid genetic instruments and the limited coverage of the NMR platform, we are unable to determine with any certainty which specific classes of fatty acids may be driving these associations.

Our analyses featured evaluating the effect of genetic liability to CRC on circulating metabolites across repeated measures in the ALSPAC cohort. The mean ages at the time of the repeated measures were 8y, 16y, 18y, and 25y, representing childhood, early adolescence, late adolescence, and young adulthood respectively, and therefore individuals in this cohort are unlikely to be taking metabolite-altering medication such as statins, and unlikely to have CRC. The strongest evidence for an effect of liability to CRC on metabolite levels was seen in late adolescence. The reason for this remains unclear. It is possible that this represents a true biological phenomenon if late adolescence is a critical window in CRC development or metabolite variability, which may be likely given the limited variance in metabolite levels at the later age of 25y (ST2). The lack of an effect at the younger ages could be explained by the fact that the CRC GRS may capture many key life events or experiences which could impact the metabolome (e.g., initiation of smoking, higher category of BMI reached, educational attainment level set, etc) but may not have yet happened at younger ages, thus obscuring an effect of genetic liability to CRC on the metabolome. Our results suggest that puberty could be important, with an effect seen seemingly particularly at the end of puberty. Repeating our analysis with sex-stratified data may aid in determining whether this is likely to be the case; sex-stratified GWAS for metabolites are not currently available to replicate such analyses. An alternative explanation is selection bias due to loss of follow-up, leading to a change in sample characteristics over time.

Another key finding in the reverse MR analysis was that genetic liability to CRC was associated with increased levels of total cholesterol, VLDL-cholesterol, LDL-cholesterol, and apolipoprotein B, though we find little evidence for a causal effect of these traits on risk of CRC in the forward MR, replicating previous forward MR analyses for total and LDL-cholesterol.^9,70–72^ This suggests that these traits may either be only predictive of (i.e., non-causal for) later CRC development, or may be influenced by the development of CRC and could have diagnostic or predictive potential. Given that the participants in the ALSPAC cohort are many decades younger than the average age of diagnosis for CRC (mean age 25 years in the latest repeated measure analysed in ALSPAC; whereas the median age at diagnosis of CRC is 64 years),^73^ the former seems the most likely scenario. Previous conventional observational studies have presented conflicting results when investigating the association between measures of cholesterol and CRC risk with some finding an inverse association and others a positive association, possibly reflecting residual confounding in conventional observational analyses.^74–80^ Previous MR studies have had similar findings to our forward MR analysis, in that there seems to be little evidence for a causal effect of cholesterol on CRC development.^70–72^ One possible explanation for how circulating levels of total cholesterol, VLDL-cholesterol, LDL-cholesterol and apolipoprotein B could predict (without necessarily causing) future CRC development could be linked to diet. A previous MR analysis suggested an effect of increased BMI on several measures of circulating cholesterol.^9^ Consuming a diet which is high in fat may increase CRC risk both through and possibly independently of adiposity, alongside increasing levels of circulating cholesterol.^81–86^ The potential for lipoprotein or apolipoprotein lipid measures in future CRC risk prediction should be further investigated.

Our analyses stratified by anatomical subsite highlighted fatty acids as being affected by genetic liability to colon and proximal colon cancer, with the forward MR confirming that fatty acid traits may be particularly important in the development of these subsites of CRC as well as distal colon cancer.

In our forward MR analyses we were unable to replicate the findings of three previous MR studies which found evidence for a causal effect of circulating linoleic acid levels on CRC development in terms of strength of evidence, though the direction of the effect estimate was similar to previous studies.^87–89^ This is surprising as all three previous analyses had a much smaller sample size than that included in our analysis (the largest had sample size of 24,748 for exposure vs 118,466 presently; and 11,016 cases and 13,732 controls for outcome vs 52,775 cases and 45,940 controls presently). Our analysis using updated genetic instruments to proxy fatty acids may be more successful in accurately instrumenting heterogenous phenotypes such as metabolite levels compared with previous analyses. All other findings in our forward MR analysis are consistent with previous MR studies where they exist.^70–72^

### Limitations

The limitations of this study include firstly the relatively small sample size included in the ALSPAC analysis, which may have implications for power and precision. Secondly, our analyses involving genetic instruments for CRC liability may have suffered from horizontal pleiotropy, even after excluding genetic variants in or near the *FADS* gene. Thirdly, our analyses were mostly restricted to white Europeans, which limits the generalisability of our findings to other populations. Fourthly, our analysis would benefit from being repeated with sex-stratified data, although such GWAS results for metabolites are not currently available. Fifthly, for our forward MR analysis, we used the UK Biobank for our exposure data. The UK Biobank has a median age of 58 at the time these measurements were taken, meaning statin use may be widespread in this population, which could be attenuating our effect estimates. Future work could attempt to replicate our analysis in a population with lower prevalence of statins intake. Finally, we included only metabolites measured using NMR. Confirming whether our results replicate using metabolite data measured with an alternative method would strengthen our findings.

### Conclusions

Our analysis provides evidence that genetic liability to CRC alters levels of metabolites at certain ages, some of which may have a causal role in CRC development. Further investigating the role of polyunsaturated fatty acids in CRC risk and circulating cholesterol in CRC prediction may be promising avenues for future research.

## Supplementary Material

1

Supplement 1

Supplement 2

## Data Availability

Individual-level ALSPAC data are available following an application. This process of managed access is detailed at www.bristol.ac.uk/alspac/researchers/access. Cohort details and data descriptions for ALSPAC are publicly available at the same web address. Summary-level GWAS data used in this study from UK Biobank are publicly available without the need for application through the MR-Base platform, which is accessible at http://www.mrbase.org/. The summary-level GWAS data for CRC used in this study are available following an application to GECCO (managed access).
