# Supplementary figures and images for: Identifying metabolic features of colorectal cancer liability using Mendelian randomization
Source: medRxiv. 2023 Nov 9:2023.03.10.23287084. Originally published 2023 Mar 10. Preprint. [Version 4] doi: 10.1101/2023.03.10.23287084 (PMC10029059; doi:10.1101/2023.03.10.23287084)

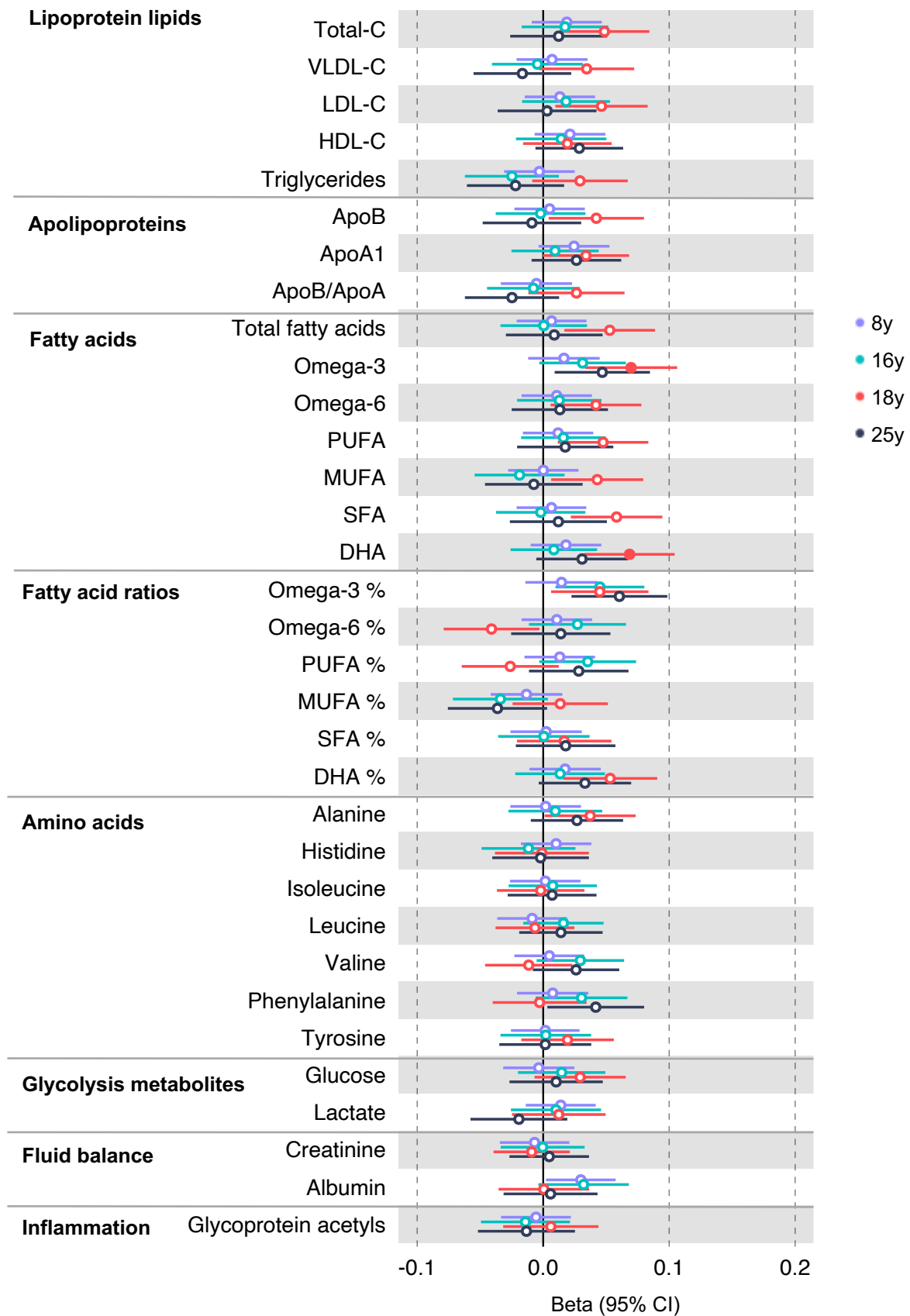

Supplement: Supplement 1 — Figure 2-figure supplement 1: Associations of genetic liability to adult colon cancer with clinically validated metabolic traits at different early life stages among ALSPAC offspring (age 8y, 16y, 18y, and 25y). Estimates shown are beta coefficients representing the SD difference in metabolic trait per doubling of genetic liability to colon cancer (purple, 8y; turquoise, 16y; red, 18y; black, 25y). Filled point estimates are those that pass a Benjamini–Hochberg FDR multiple-testing correction (FDR < 0.05). [file media-1.pdf]

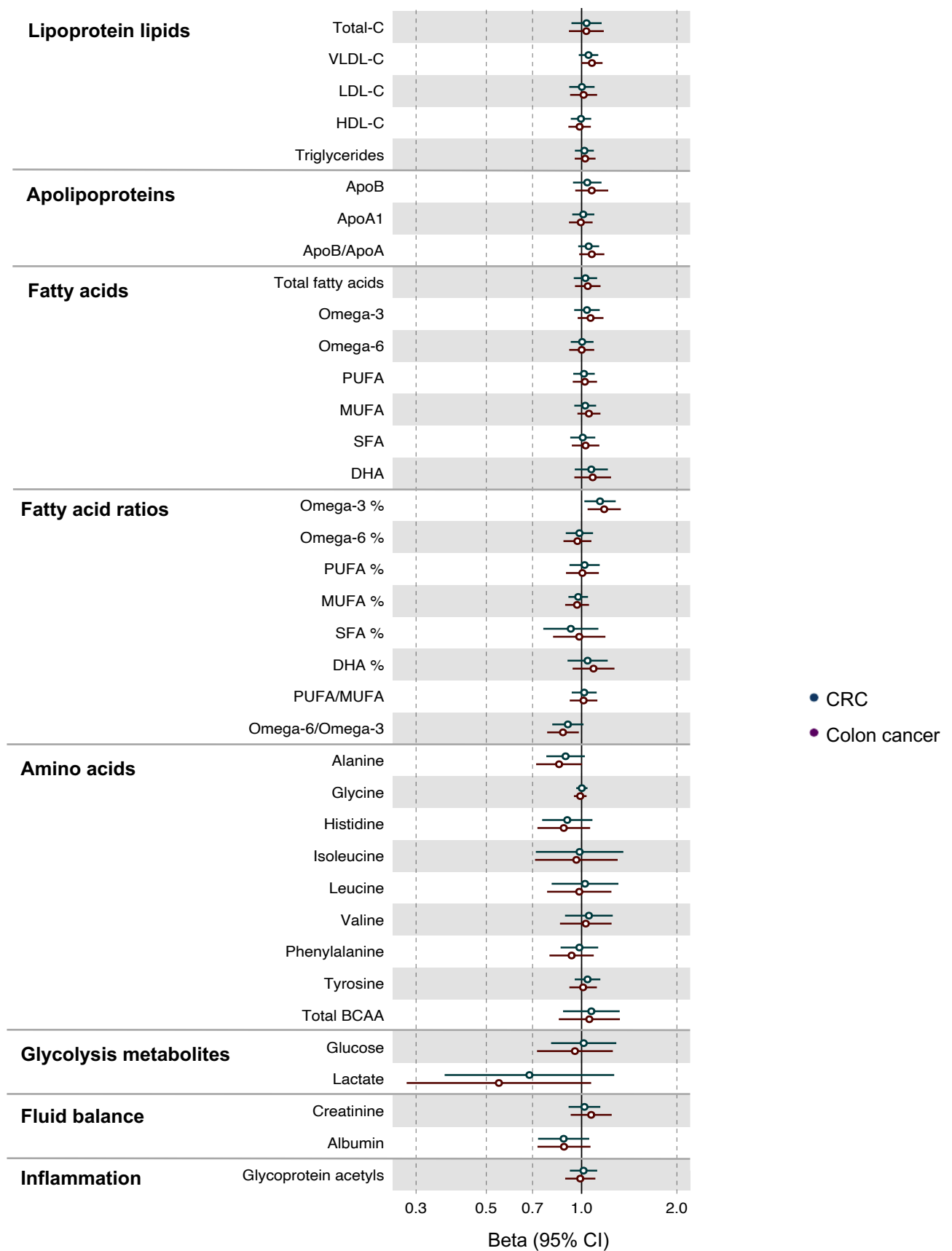

Supplement: Supplement 2 — Figure 2-figure supplement 2: Associations of genetic liability to proximal colon cancer with clinically validated metabolic traits at different early life stages among ALSPAC offspring (age 8y, 16y, 18y, and 25y). Estimates shown are beta coefficients representing the SD difference in metabolic trait per doubling of genetic liability to proximal colon cancer (purple, 8y; turquoise, 16y; red, 18y; black, 25y). Filled point estimates are those that pass a Benjamini–Hochberg FDR multiple-testing correction (FDR < 0.05). [file media-2.pdf]

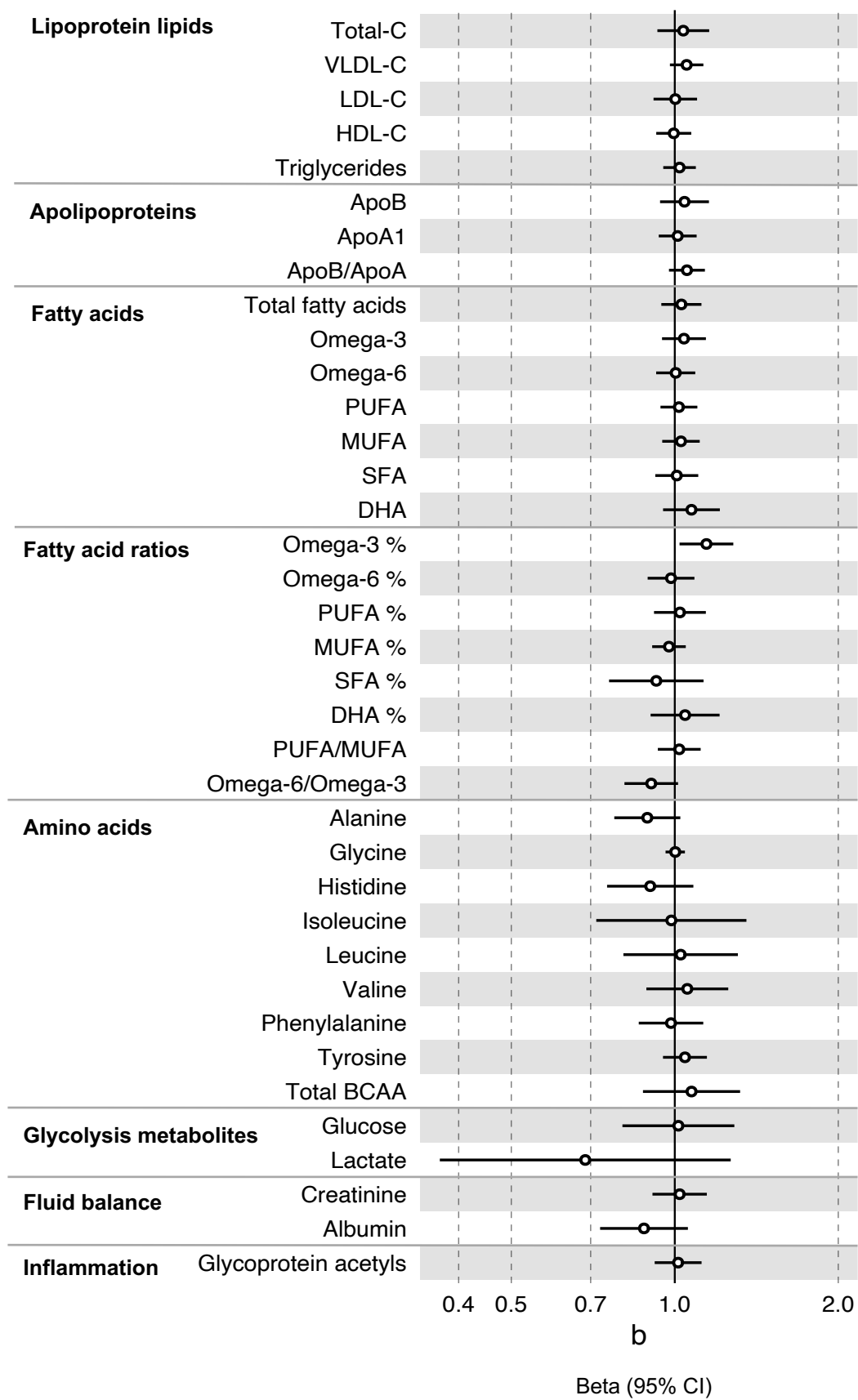

Supplement: Supplement 3 — Figure 2-figure supplement 3: Associations of genetic liability to distal colon cancer with clinically validated metabolic traits at different early life stages among ALSPAC offspring (age 8y, 16y, 18y, and 25y). Estimates shown are beta coefficients representing the SD difference in metabolic trait per doubling of genetic liability to distal colon cancer (purple, 8y; turquoise, 16y; red, 18y; black, 25y). Filled point estimates are those that pass a Benjamini–Hochberg FDR multiple-testing correction (FDR < 0.05). [file media-3.pdf]

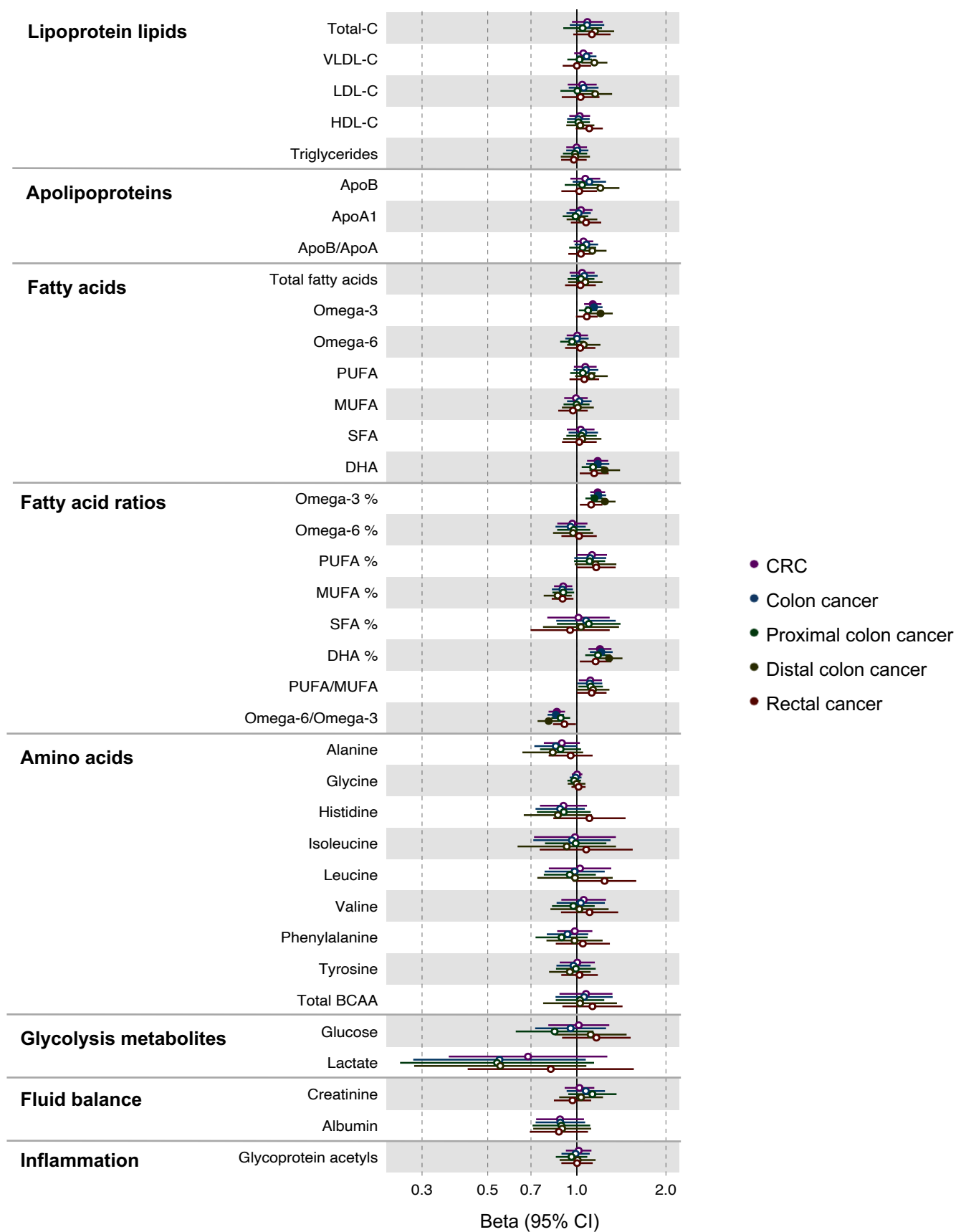

Supplement: Supplement 4 — Figure 2-figure supplement 4: Associations of genetic liability to rectal cancer with clinically validated metabolic traits at different early life stages among ALSPAC offspring (age 8y, 16y, 18y, and 25y). Estimates shown are beta coefficients representing the SD difference in metabolic trait per doubling of genetic liability to rectal cancer (purple, 8y; turquoise, 16y; red, 18y; black, 25y). Filled point estimates are those that pass a Benjamini–Hochberg FDR multiple-testing correction (FDR < 0.05). [file media-4.pdf]

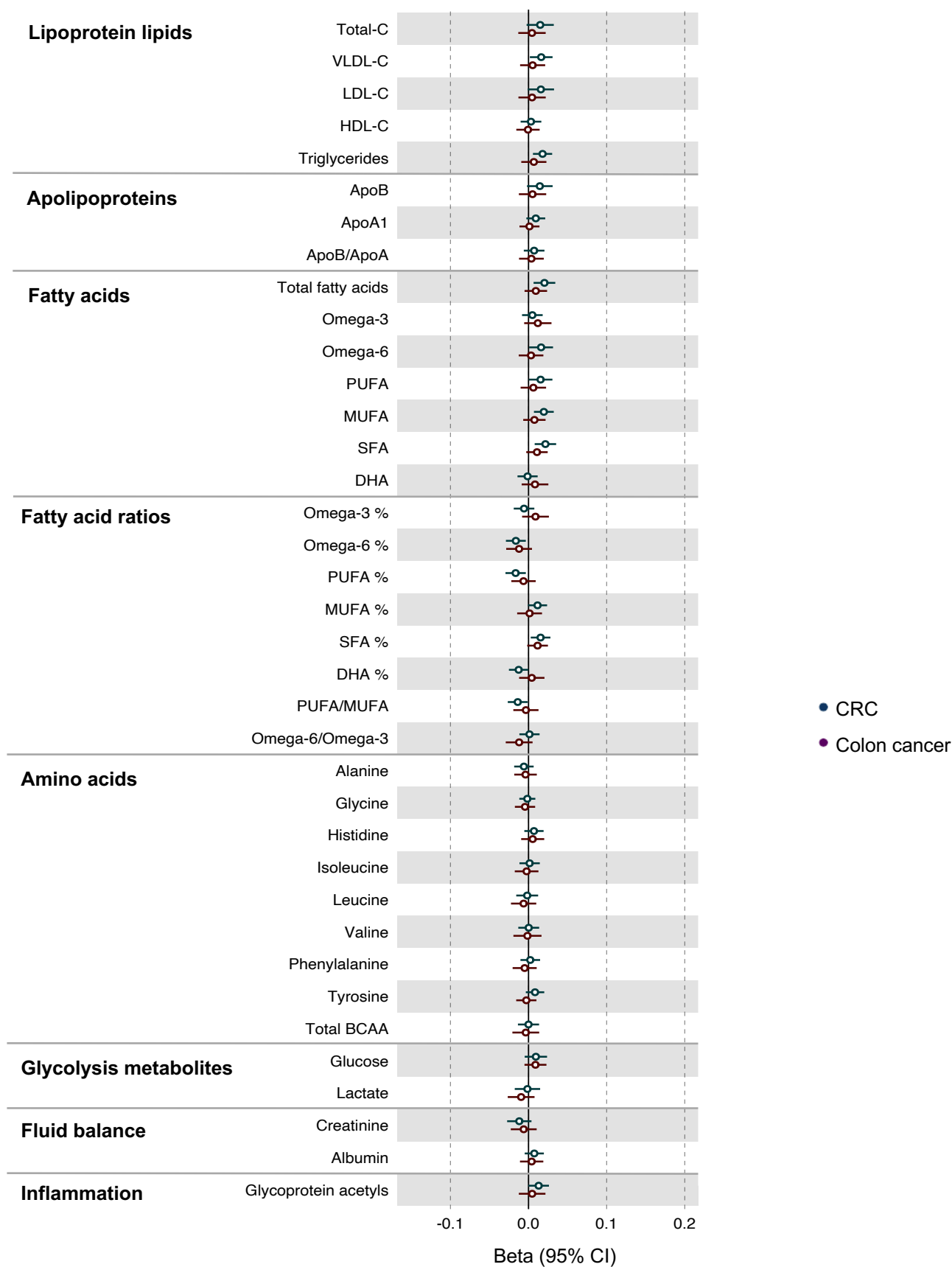

Supplement: Supplement 5 — Figure 2-figure supplement 5: Associations of genetic liability to adult colorectal cancer (excluding rs174533) with clinically validated metabolic traits at different early life stages among ALSPAC offspring (age 8y, 16y, 18y, and 25y). Estimates shown are beta coefficients representing the SD difference in metabolic trait per doubling of genetic liability to colorectal cancer (purple, 8y; turquoise, 16y; red, 18y; black, 25y). Filled point estimates are those that pass a Benjamini–Hochberg FDR multiple-testing correction (FDR < 0.05). [file media-5.pdf]

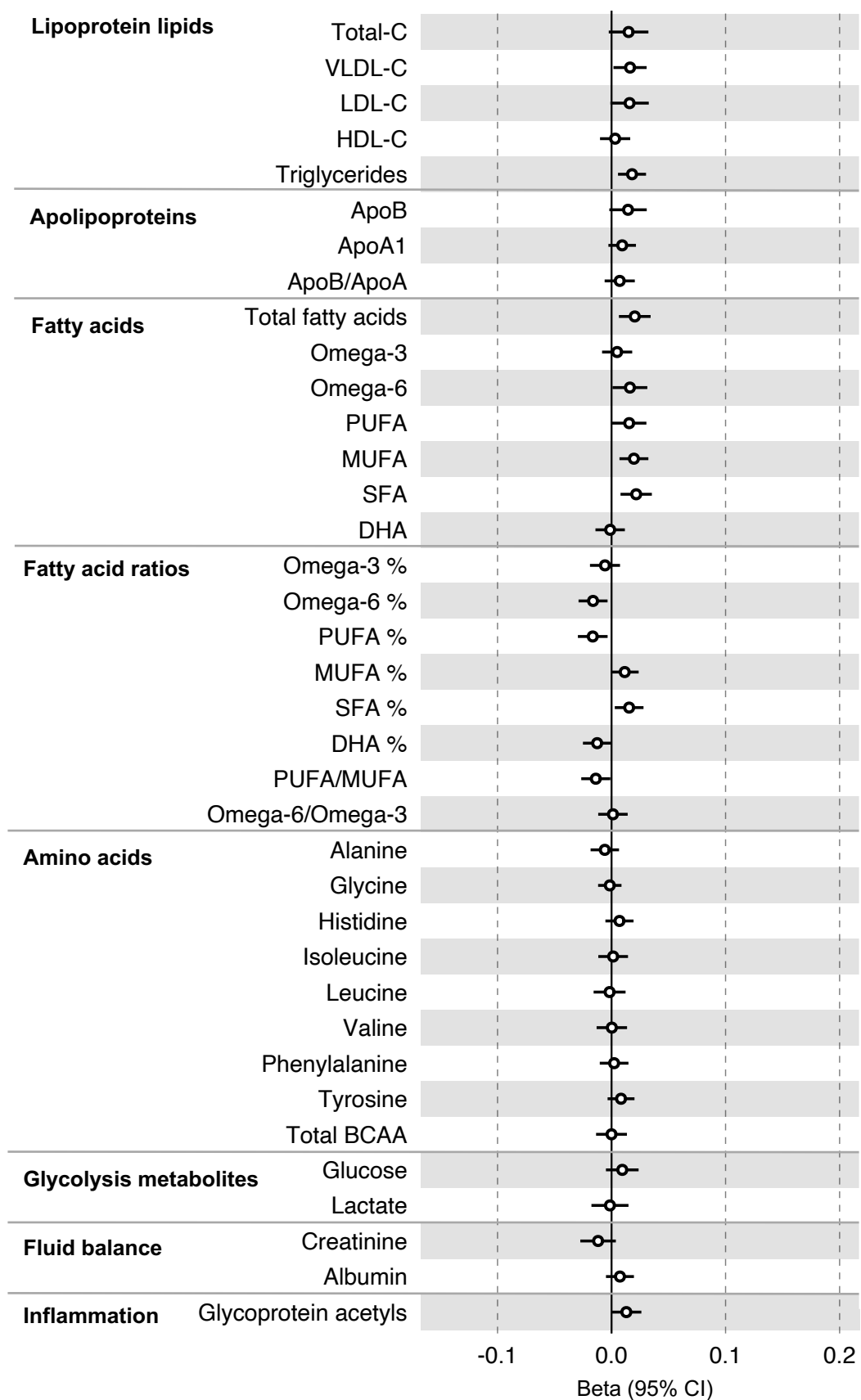

Supplement: Supplement 6 — Figure 2-figure supplement 6: Associations of genetic liability to adult colon cancer (excluding rs174535) with clinically validated metabolic traits at different early life stages among ALSPAC offspring (age 8y, 16y, 18y, and 25y). Estimates shown are beta coefficients representing the SD difference in metabolic trait per doubling of genetic liability to colorectal cancer cancer (purple, 8y; turquoise, 16y; red, 18y; black, 25y). Filled point estimates are those that pass a Benjamini–Hochberg FDR multiple-testing correction (FDR < 0.05). [file media-6.pdf]

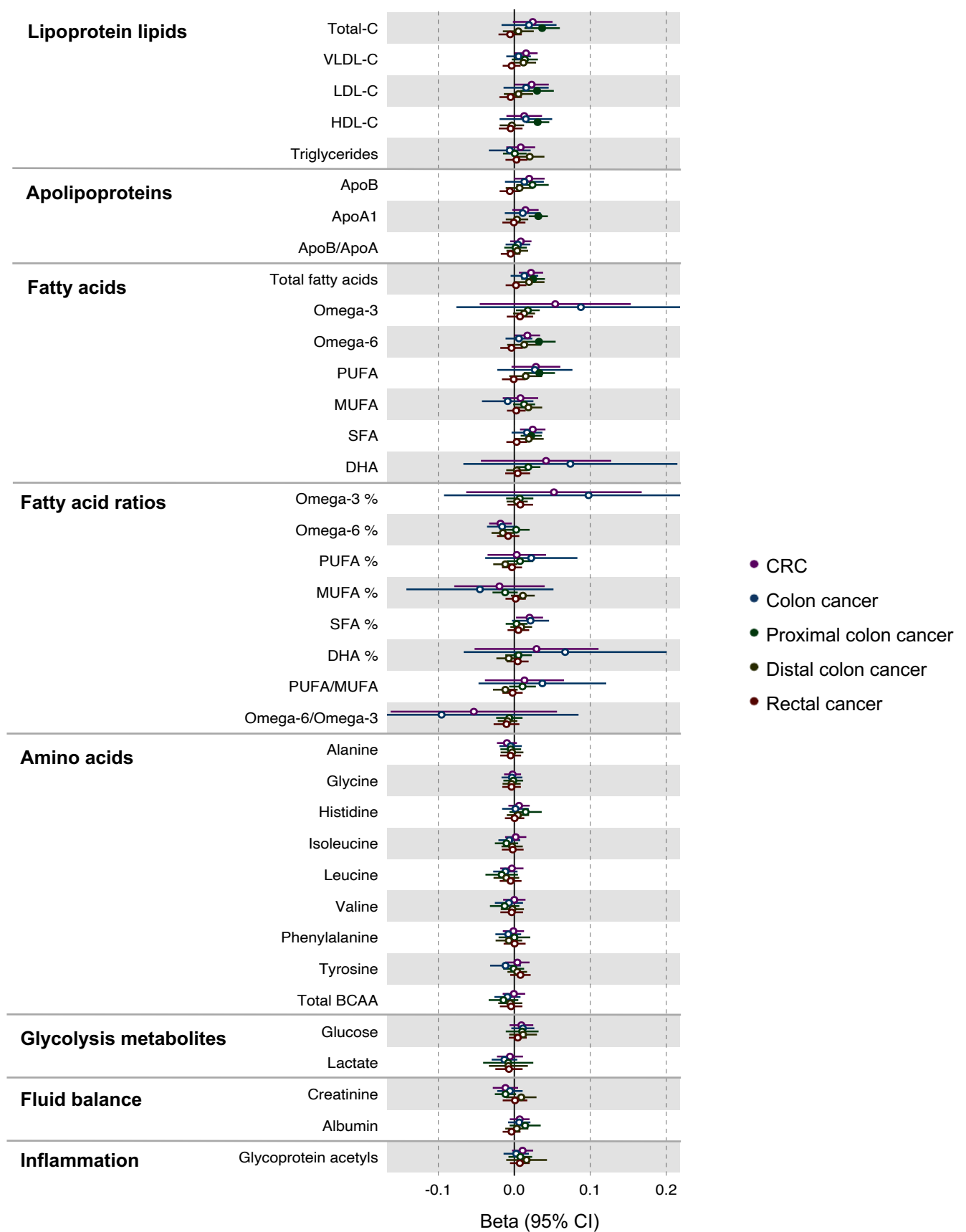

Supplement: Supplement 7 — Figure 3-figure supplement 1: Associations of genetic liability to colorectal cancer with clinically validated metabolic traits in an independent sample of adults based on reverse two sample Mendelian randomization analyses. Estimates shown are beta coefficients representing the SD-unit difference in metabolic trait per doubling of liability to colorectal cancer by site (colorectal, colon, distal colon, proximal colon and rectal cancer). Filled point estimates are those that pass a Benjamini–Hochberg FDR multiple-testing correction (FDR < 0.05). [file media-7.pdf]

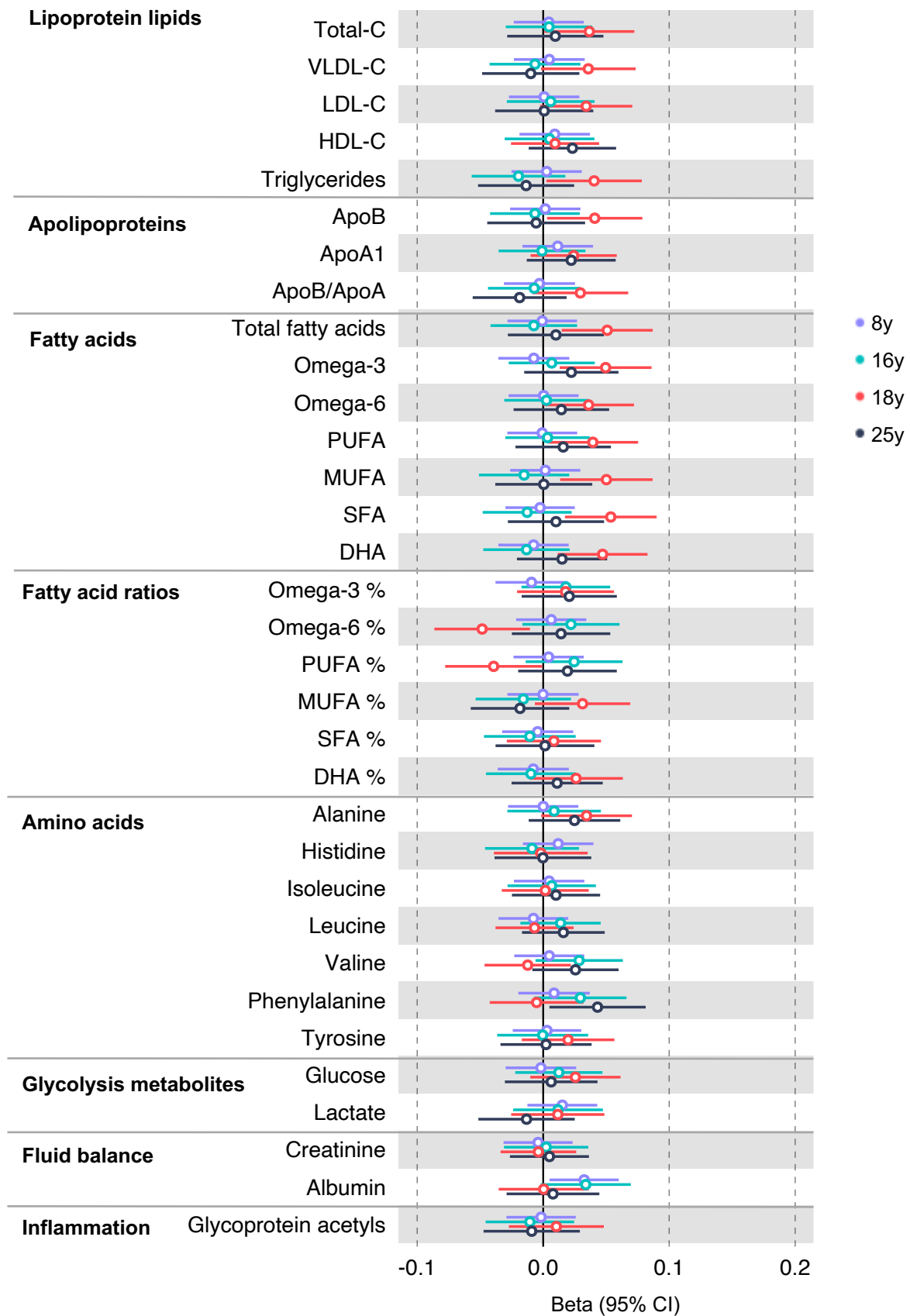

Supplement: Supplement 8 — Figure 3-figure supplement 2: Associations of genetic liability to colorectal cancer (excluding genetic variants in the FADS gene region) with clinically validated metabolic traits in an independent sample of adults based on reverse two sample Mendelian randomization analyses. Estimates shown are beta coefficients representing the SD-unit difference in metabolic trait per doubling of liability to colorectal cancer. Filled point estimates are those that pass a Benjamini–Hochberg FDR multiple-testing correction (FDR < 0.05). [file media-8.pdf]

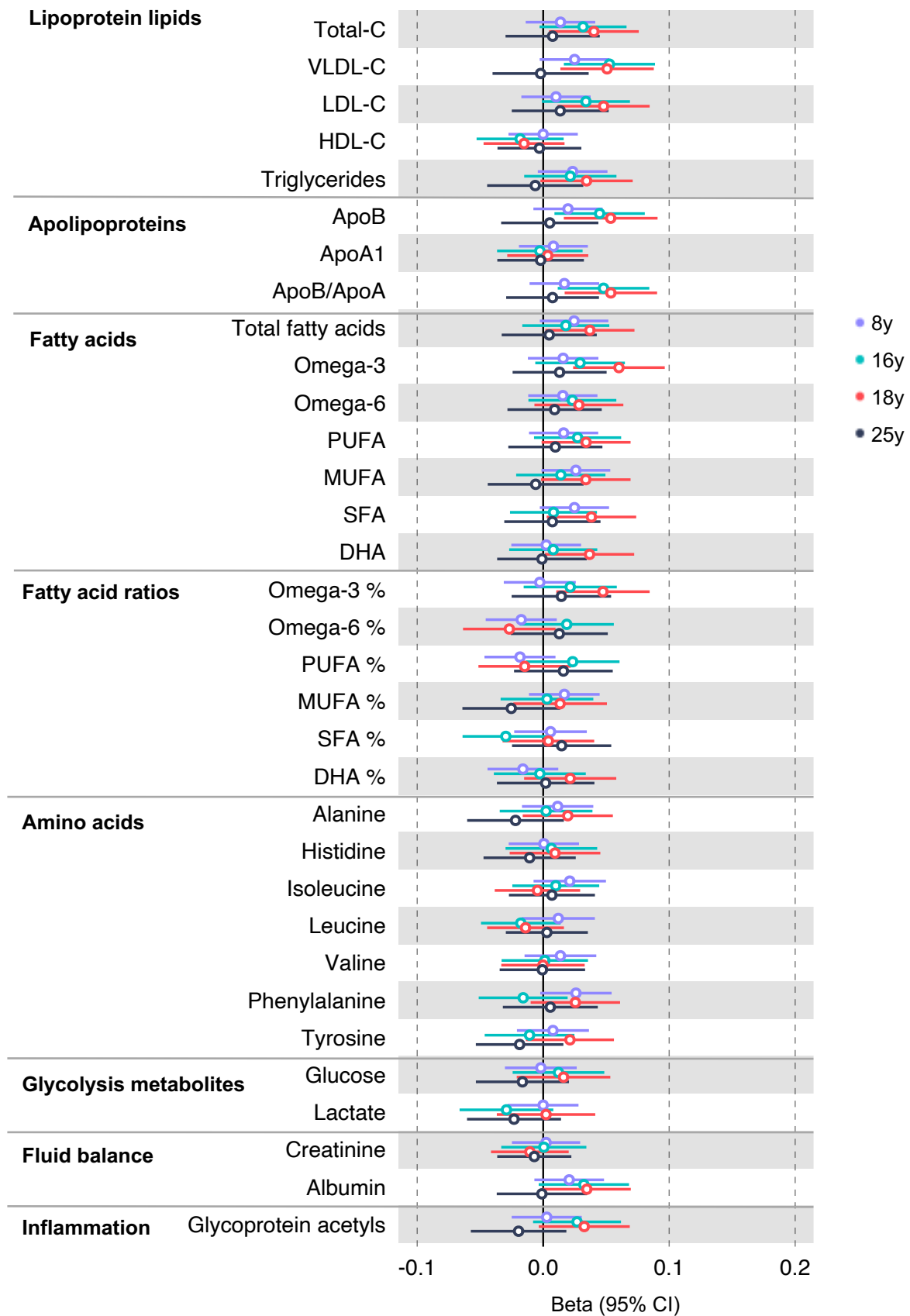

Supplement: Supplement 9 — Figure 3-figure supplement 3: Associations of genetic liability to colorectal and colon cancer with clinically validated metabolic traits in an independent sample of adults based on reverse two sample Mendelian randomization analyses with FADS variants excluded from colorectal cancer instruments. Estimates shown are beta coefficients representing the SD-unit difference in metabolic trait per doubling of liability to colorectal cancer by site (colorectal, colon). Filled point estimates are those that pass a Benjamini–Hochberg FDR multiple-testing correction (FDR < 0.05). [file media-9.pdf]

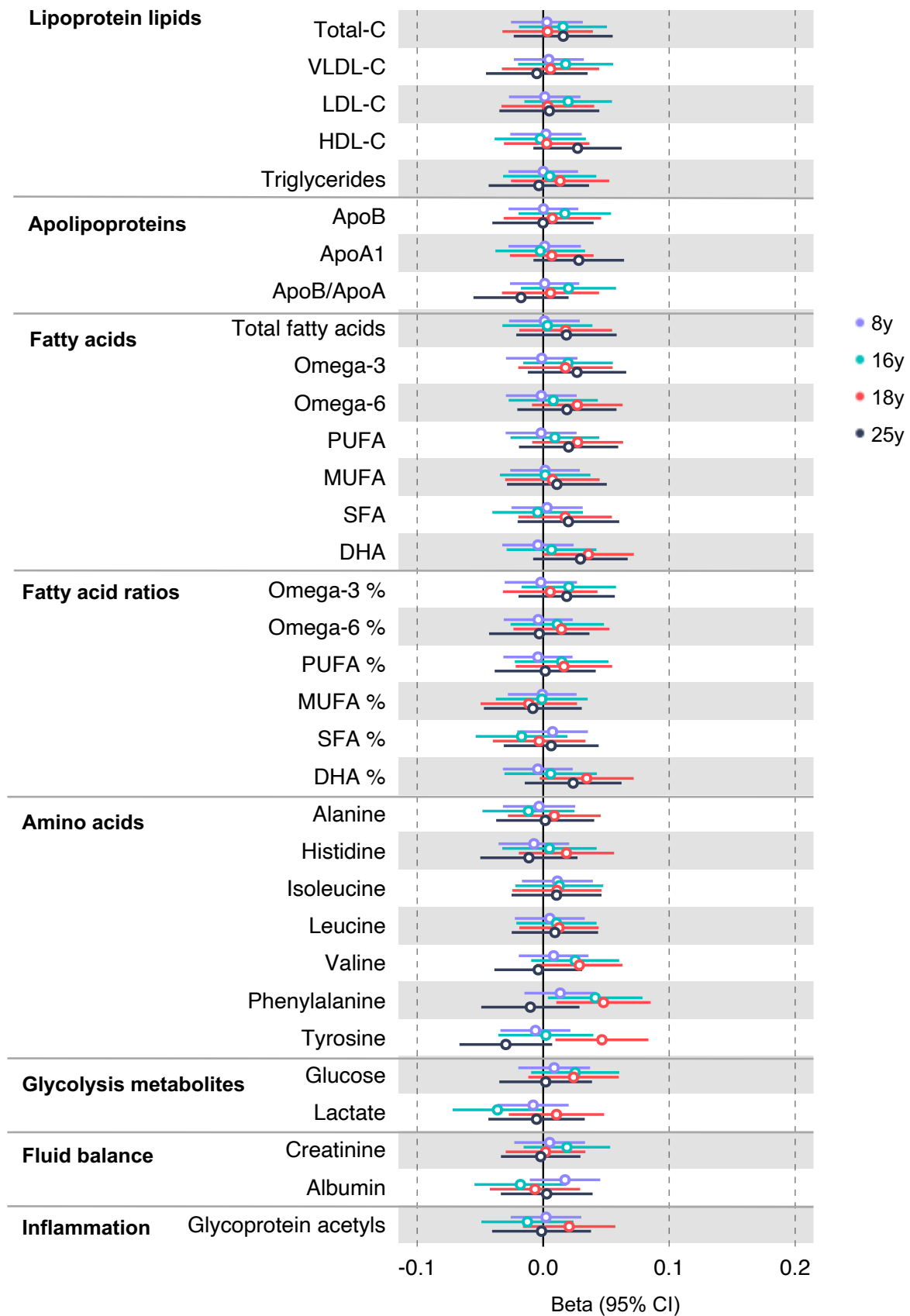

Supplement: Supplement 10 — Figure 4-figure supplement 1: Associations of clinically validated metabolites with colorectal cancer by site (colorectal, colon, distal colon, proximal colon and rectal cancer) based on conventional (forward) two sample Mendelian randomization analyses. Estimates shown are ORs for colorectal cancer per SD metabolite. Filled point estimates are those that pass a Benjamini–Hochberg FDR multiple-testing correction (FDR < 0.05). [file media-10.pdf]

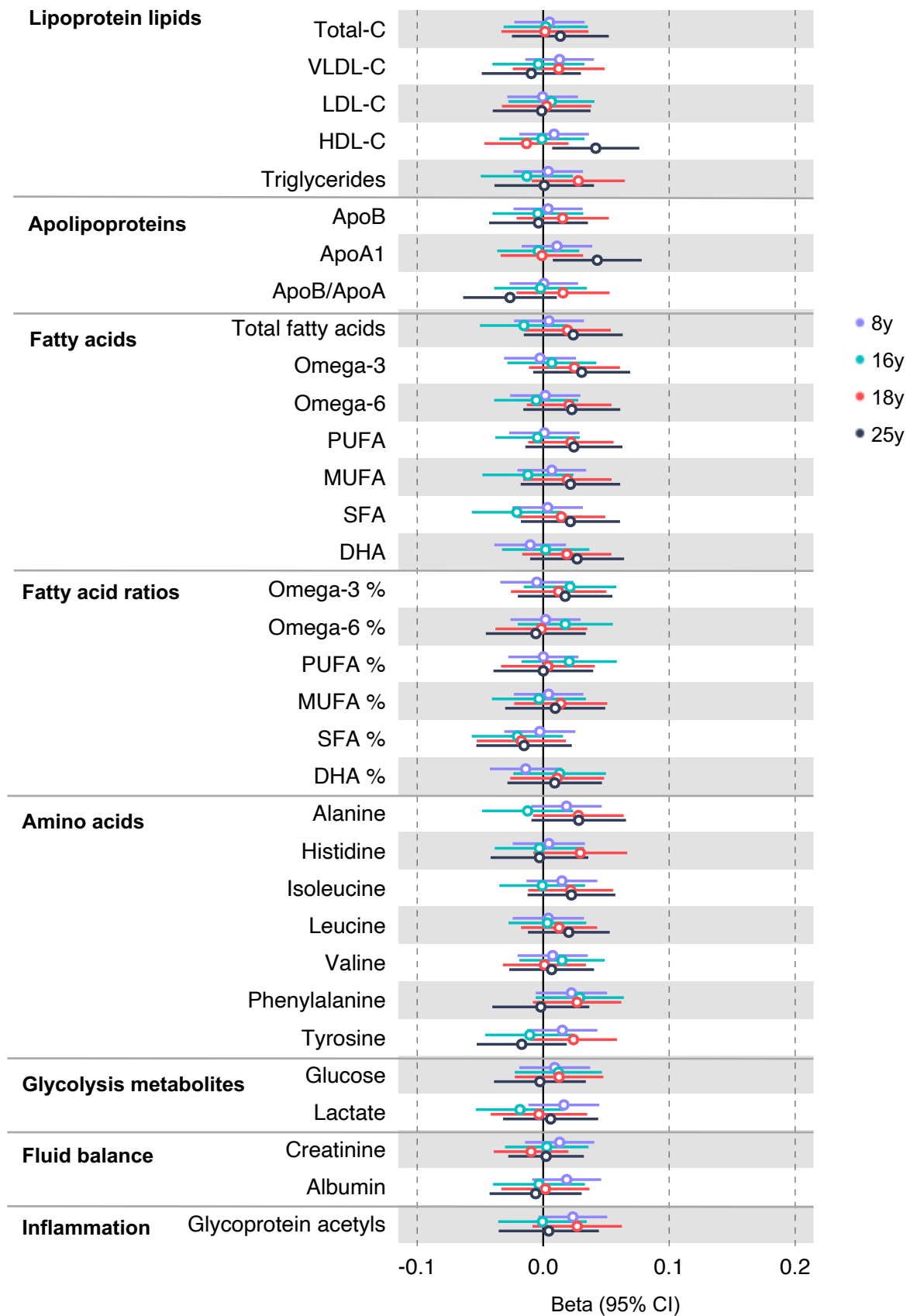

Supplement: Supplement 11 [file media-11.pdf]

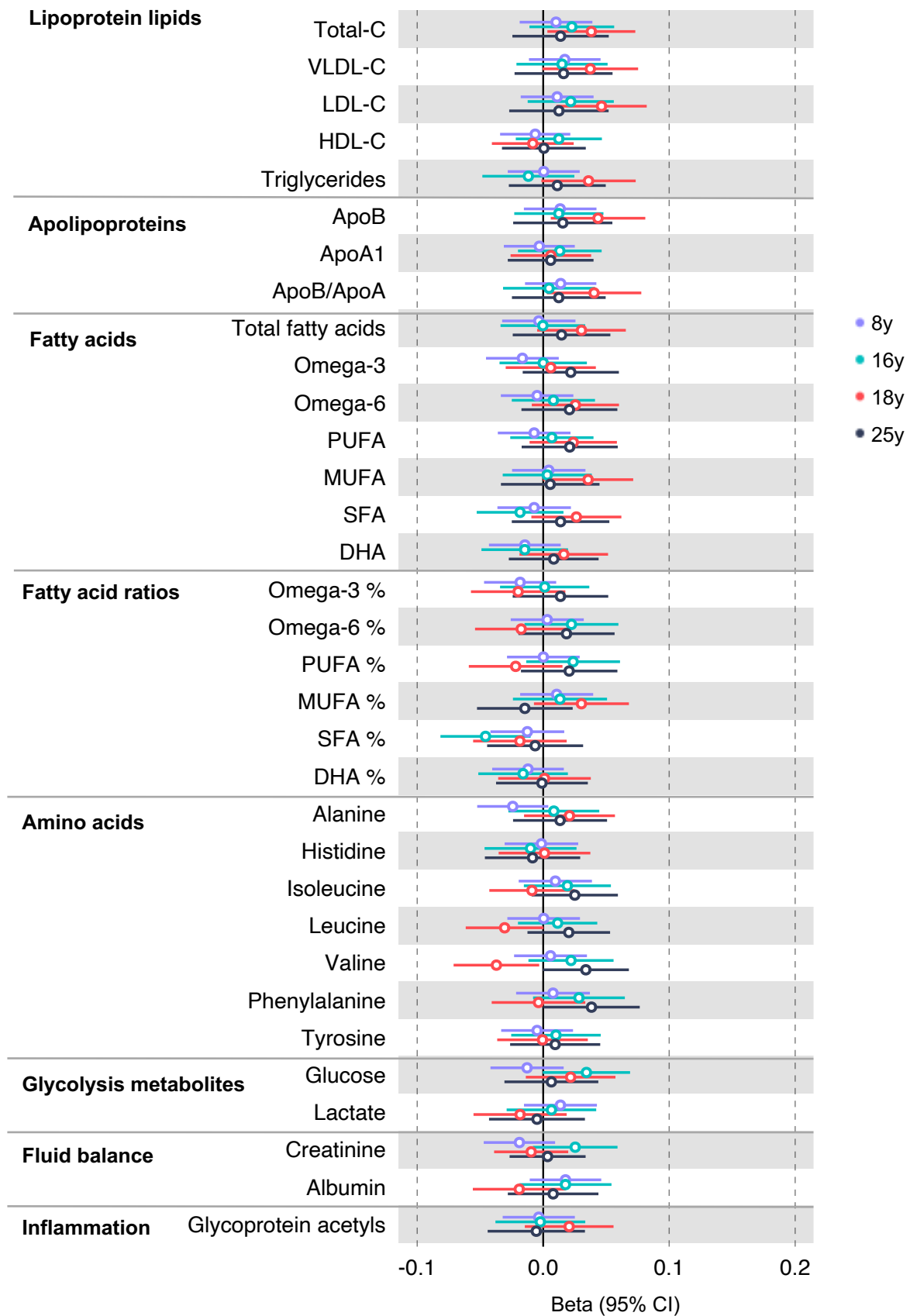

Supplement: Supplement 12 [file media-12.pdf]
