# Supplementary material for: Identifying metabolic features of colorectal cancer liability using Mendelian randomization
Source: medRxiv. 2023 Jul 17:2023.03.10.23287084. Originally published 2023 Mar 10. Preprint. [Version 2] doi: 10.1101/2023.03.10.23287084 (PMC10029059; doi:10.1101/2023.03.10.23287084)
Supplement: Supplementary file 1 [file media-3.pdf]

STROBE-MR checklist of recommended items to address in reports of Mendelian randomization studies[1 2]

Item No.	Section		Checklist item	Page No.	Relevant text from manuscript
1	**TITLE and ABSTRACT**		Indicate Mendelian randomization (MR) as the study's design in the title and/or the abstract if that is a main purpose of the study	1-2	Identifying metabolic features of colorectal cancer liability using Mendelian randomization<br><br>Two-sample reverse Mendelian randomization (MR) analyses investigating CRC liability (52,775 cases, 45,940 controls) and metabolites measured in a random subset of UK Biobank participants
	**INTRODUCTION**				
2	**Background**		Explain the scientific background and rationale for the reported study. What is the exposure? Is a potential causal relationship between exposure and outcome plausible? Justify why MR is a helpful method to address the study question	4	One potential source of biomarkers for CRC is the circulating metabolome, which offers dynamic insight into cellular processes and disease states.<br><br>Given the suspected importance of the circulating metabolome in CRC development, employing both reverse MR and conventional forward MR for metabolites in the same study may be an efficient approach for revealing causal and predictive biomarkers for CRC.
3	**Objectives**		State specific objectives clearly, including pre-specified causal hypotheses (if any). State that MR is a method that, under specific assumptions, intends to estimate causal effects	4	As germline genetic variants are theoretically randomised between generations, using these variants in place of direct exposure/outcome measures should be less prone to bias and confounding than conventional analyses undertaken in an observational context. Additionally, as the variants are fixed at conception, there should be less bias from reverse causation.
	**METHODS**				
4	**Study design and data sources**		Present key elements of the study design early in the article. Consider including a table listing sources of data for all phases of the study. For each data source contributing to the analysis, describe the following:		
		a)	Setting: Describe the study design and the underlying population, if possible. Describe the setting, locations, and relevant dates, including periods of recruitment, exposure, follow-up, and data collection, when available.	5-7	Detailed descriptions of GECCO, ALSPAC and UK Biobank cohorts

		b)	Participants: Give the eligibility criteria, and the sources and methods of selection of participants. Report the sample size, and whether any power or sample size calculations were carried out prior to the main analysis	5-7	Detailed descriptions of GECCO, ALSPAC and UK Biobank cohorts
		c)	Describe measurement, quality control and selection of genetic variants	5-7	Detailed descriptions of GECCO, ALSPAC and UK Biobank cohorts
		d)	For each exposure, outcome, and other relevant variables, describe methods of assessment and diagnostic criteria for diseases	5-7	Detailed descriptions of GECCO, ALSPAC and UK Biobank cohorts
		e)	Provide details of ethics committee approval and participant informed consent, if relevant	6	Included in ALSPAC study description
5	**Assumptions**		Explicitly state the three core IV assumptions for the main analysis (relevance, independence and exclusion restriction) as well assumptions for any additional or sensitivity analysis	4-5	
6	**Statistical methods: main analysis**		Describe statistical methods and statistics used		
		a)	Describe how quantitative variables were handled in the analyses (i.e., scale, units, model)	9	Prior to GWAS, all metabolite measures were standardised and normalised using rank-based inverse normal transformation.<br>To aid interpretations, estimates were multiplied by 0.693 ($\log_e 2$) to reflect SD-unit differences in metabolites per doubling of genetic liability to CRC
		b)	Describe how genetic variants were handled in the analyses and, if applicable, how their weights were selected	9	Up to 3 statistical methods were used to generate reverse MR estimates of the effect of CRC liability on circulating metabolites using the TwoSampleMR package[58]: random-effects inverse variance weighted (IVW), weighted-median, and weighted-mode
		c)	Describe the MR estimator (e.g. two-stage least squares, Wald ratio) and related statistics. Detail the included covariates and, in case of two-sample MR, whether the same covariate set was used for adjustment in the two samples	7,9	As above, and adjusted for age, sex, genotyping platform, and genomic principal components as described previously
		d)	Explain how missing data were addressed	NA	NA
		e)	If applicable, indicate how multiple testing was addressed		
7	**Assessment of assumptions**		Describe any methods or prior knowledge used to assess the assumptions or justify their validity	9	The Benjamini-Hochberg method was used to adjust P-values for multiple testing and an adjusted P-value of <0.05 was used as a

					heuristic for evidence for association given current sample sizes
8	**Sensitivity analyses and additional analyses**		Describe any sensitivity analyses or additional analyses performed (e.g. comparison of effect estimates from different approaches, independent replication, bias analytic techniques, validation of instruments, simulations)	9	As above.
9	**Software and pre-registration**				
		a)	Name statistical software and package(s), including version and settings used	7,10	using the TwoSampleMR package<br>MR analyses were performed in R version 4.0.3. and GRS analyses in Stata 16.1 (StataCorp, College Station, Texas, USA). The ggforestplot R package was used to generate results visualisations.
		b)	State whether the study protocol and details were pre-registered (as well as when and where)	NA	NA
	**RESULTS**				
10	**Descriptive data**				
		a)	Report the numbers of individuals at each stage of included studies and reasons for exclusion. Consider use of a flow diagram	Fig 1	
		b)	Report summary statistics for phenotypic exposure(s), outcome(s), and other relevant variables (e.g. means, SDs, proportions)	ST2	
		c)	If the data sources include meta-analyses of previous studies, provide the assessments of heterogeneity across these studies	NA	NA
		d)	For two-sample MR:<br>i. Provide justification of the similarity of the genetic variant-exposure associations between the exposure and outcome samples<br>ii. Provide information on the number of individuals who overlap between the exposure and outcome studies		No overlap between studies and same underlying populations used
11	**Main results**				
		a)	Report the associations between genetic variant and exposure, and between genetic variant and outcome, preferably on an interpretable scale	ST5 and ST9	

		b)	Report MR estimates of the relationship between exposure and outcome, and the measures of uncertainty from the MR analysis, on an interpretable scale, such as odds ratio or relative risk per SD difference	11	E.g.: In subsite stratified analyses, there was strong evidence for a causal effect of genetic liability to proximal colon cancer on several traits, including total fatty acids (SD change per doubling of liability = 0.02, 95% CI = 0.01 to 0.04) and omega-6 fatty acids (SD change per doubling of liability = 0.03, 95% CI = 0.01 to 0.05).
		c)	If relevant, consider translating estimates of relative risk into absolute risk for a meaningful time period	NA	NA
		d)	Consider plots to visualize results (e.g. forest plot, scatterplot of associations between genetic variants and outcome versus between genetic variants and exposure)	Fig 2-4	
12	**Assessment of assumptions**				
		a)	Report the assessment of the validity of the assumptions	12	These estimates were overlapping with variable precision in MR sensitivity models
		b)	Report any additional statistics (e.g., assessments of heterogeneity across genetic variants, such as I^2, Q statistic or E-value)	NA	NA
13	**Sensitivity analyses and additional analyses**				
		a)	Report any sensitivity analyses to assess the robustness of the main results to violations of the assumptions	11-12	These estimates were overlapping with variable precision in MR sensitivity models<br>When SNPs in the *FADS* gene region were excluded, there was little evidence for a causal effect of any metabolite investigated on CRC risk based on the predetermined FDR-P cut of off < 0.05
		b)	Report results from other sensitivity analyses or additional analyses	NA	NA
		c)	Report any assessment of direction of causal relationship (e.g., bidirectional MR)		Throughout, by comparing reverse and forward MRs
		d)	When relevant, report and compare with estimates from non-MR analyses	NA	NA
		e)	Consider additional plots to visualize results (e.g., leave-one-out analyses)	NA	NA

	**DISCUSSION**			
14	**Key results**	Summarize key results with reference to study objectives	13-15	E.g. . Previous conventional observational studies have presented conflicting results when investigating the association between measures of cholesterol and CRC risk with some finding an inverse association and others a positive association, possibly reflecting residual confounding in conventional observational analyses
15	**Limitations**	Discuss limitations of the study, taking into account the validity of the IV assumptions, other sources of potential bias, and imprecision. Discuss both direction and magnitude of any potential bias and any efforts to address them	15	Limitations paragraph
16	**Interpretation**			
	a)	Meaning: Give a cautious overall interpretation of results in the context of their limitations and in comparison with other studies	16	Our analysis provides evidence for an important role of the circulating metabolome in CRC development and prediction. Further investigating the role of polyunsaturated fatty acids in CRC development and circulating cholesterol in CRC prediction may be promising avenues for future research.
	b)	Mechanism: Discuss underlying biological mechanisms that could drive a potential causal relationship between the investigated exposure and the outcome, and whether the gene-environment equivalence assumption is reasonable. Use causal language carefully, clarifying that IV estimates may provide causal effects only under certain assumptions	14	One possible explanation for how circulating levels of total cholesterol, VLDL-cholesterol, LDL-cholesterol and apolipoprotein B could predict (without necessarily causing) future CRC development could be linked to diet. A previous MR analysis suggested an effect of increased BMI on several measures of circulating cholesterol.Consuming a diet which is high in fat may increase CRC risk both through and possibly independently of adiposity, alongside increasing levels of circulating cholesterol
	c)	Clinical relevance: Discuss whether the results have clinical or public policy relevance, and to what extent they inform effect sizes of possible interventions	16	Further investigating the role of polyunsaturated fatty acids in CRC development and circulating cholesterol in CRC prediction may be promising avenues for future research.
17	**Generalizability**	Discuss the generalizability of the study results (a) to other populations, (b) across other exposure periods/timings, and (c) across other levels of exposure	15	Our analyses were mostly restricted to white Europeans, which limits the generalisability of our findings to other populations

	**OTHER INFORMATION**			
18	**Funding**	Describe sources of funding and the role of funders in the present study and, if applicable, sources of funding for the databases and original study or studies on which the present study is based	25	Funding and acknowledgement sections<br>The funders had no role in study design, data collection and analysis, decision to publish, or preparation of the manuscript.
19	**Data and data sharing**	Provide the data used to perform all analyses or report where and how the data can be accessed, and reference these sources in the article. Provide the statistical code needed to reproduce the results in the article, or report whether the code is publicly accessible and if so, where	Supplementary tables	
20	**Conflicts of Interest**	All authors should declare all potential conflicts of interest	NA	NA

This checklist is copyrighted by the Equator Network under the Creative Commons Attribution 3.0 Unported (CC BY 3.0) license.

1. Skrivankova VW, Richmond RC, Woolf BAR, Yarmolinsky J, Davies NM, Swanson SA, et al. Strengthening the Reporting of Observational Studies in Epidemiology using Mendelian Randomization (STROBE-MR) Statement. JAMA. 2021;under review.

2. Skrivankova VW, Richmond RC, Woolf BAR, Davies NM, Swanson SA, VanderWeele TJ, et al. Strengthening the Reporting of Observational Studies in Epidemiology using Mendelian Randomisation (STROBE-MR): Explanation and Elaboration. BMJ. 2021;375:n2233.
